# Climate Change, Crop Yields, and Grain Quality of C_3_ Cereals: A Meta-Analysis of [CO_2_], Temperature, and Drought Effects

**DOI:** 10.3390/plants10061052

**Published:** 2021-05-24

**Authors:** Sinda Ben Mariem, David Soba, Bangwei Zhou, Irakli Loladze, Fermín Morales, Iker Aranjuelo

**Affiliations:** 1 Instituto de Agrobiotecnología (IdAB), CSIC-Gobierno de Navarra, Avda. de Pamplona 123, 31192 Mutilva, Spain; sinda.ben@csic.es (S.B.M.); david.soba@unavarra.es (D.S.); fermin.morales@csic.es (F.M.); 2 Key Laboratory of Vegetation Ecology, Institute of Grassland Science, Northeast Normal University, Ministry of Education, Changchun 130024, China; zhoubw599@nenu.edu.cn; 3 Bryan Medical Center, Bryan College of Health Sciences, Lincoln, NE 68506, USA; loladze@gmail.com

**Keywords:** cereals, yield and quality, high [CO_2_], predicted future climate, high temperature, grain quality traits, drought stress

## Abstract

Cereal yield and grain quality may be impaired by environmental factors associated with climate change. Major factors, including elevated CO_2_ concentration ([CO_2_]), elevated temperature, and drought stress, have been identified as affecting C_3_ crop production and quality. A meta-analysis of existing literature was performed to study the impact of these three environmental factors on the yield and nutritional traits of C_3_ cereals. Elevated [CO_2_] stimulates grain production (through larger grain numbers) and starch accumulation but negatively affects nutritional traits such as protein and mineral content. In contrast to [CO_2_], increased temperature and drought cause significant grain yield loss, with stronger effects observed from the latter. Elevated temperature decreases grain yield by decreasing the thousand grain weight (TGW). Nutritional quality is also negatively influenced by the changing climate, which will impact human health. Similar to drought, heat stress decreases starch content but increases grain protein and mineral concentrations. Despite the positive effect of elevated [CO_2_], increases to grain yield seem to be counterbalanced by heat and drought stress. Regarding grain nutritional value and within the three environmental factors, the increase in [CO_2_] is possibly the more detrimental to face because it will affect cereal quality independently of the region.

## 1. Introduction

Food security is threatened by the impacts of climate change on agriculture and by increasing the world population [1,2]. Actually, climate change has already slowed global agricultural productivity growth, and in a recent study, Ortiz-Bobea et al. [3] found that anthropogenic climate change (ACC) has reduced global agricultural total factor productivity since 1961 by about 21%, with a greater impact for warm regions such as Africa (−34%) than for cooler regions such as Europe and Central Asia (−7.1%). Over the next few decades, climate change is expected to affect more the world’s supply of cereal grains, impacting their quantity and quality due to the complex effects of elevated atmospheric [CO_2_] and changing temperature and rainfall patterns on crops [4]. Cereals contribute to a substantial part of the world’s plant-derived food production and comprise a majority of the crops harvested. In fact, FAO statistics show that in 2016, sugar cane had the highest production globally, followed by corn, wheat, and rice [5]. Adding to that, according to the Foreign Agricultural Service/USDA, preliminary world production in 2018 of maize, wheat, and rice was estimated at around 1076, 763, and 495 million tons, respectively [6]. Further, their nutritional quality has a significant impact on human well-being and health, especially in the developing world [7]. Thus, one of the major challenges that plant breeders are facing currently is to increase cereal grain production while taking into consideration an adequate grain nutrient content.

Numerous effects of elevated atmospheric [CO_2_] on plants have been documented through a photosynthesis-mediated CO_2_fertilization effect, including increased carbon (C) assimilation, growth, yield, and C content [8,9]. Thus, elevated [CO_2_] could enhance the concentration of photosynthesis-derived carbohydrates in grains, starch being the major component [10,11,12]. Since grains are predominantly composed of carbohydrates (mostly in the form of starch), it has been suggested that increases in starch concentrations can cause a dilution effect onother nutrients, including proteins, lipids, vitamins, and minerals. In addition, adjustments in the photosynthetic apparatus and later on the redistribution from senescing leaves to grains must be considered as the key mechanisms. Due to the different biochemistry of C_3_ and C_4_ photosynthesis, the positive effect of elevated [CO_2_] on photosynthesis is more pronounced in C_3_ crops such as wheat and rice but less notable in C_4_ crops such as maize [13]. Rising [CO_2_] is likely to lead to “globally imbalanced plant stoichiometry (relative to pre-industrial times)” [14], which in turn would “intensify the already acute problem of micronutrient malnutrition” [14], particularly regarding minerals such asFe, Zn, and I, as well asprotein (or N) [12,15,16]. Elevated [CO_2_] has been reported to decrease mineral concentrations in barley grains (−6.9%), rice grains (−7.2%), and wheat grains (−7.6%), and increasing the ratio of non-structural carbohydrates (TNC) to protein by 6–47% in grains and tubers [9]. For the grain crops barley, rice, and wheat, the reduction in protein mediated by elevated [CO_2_] was reported to be 15, 10, and 10%, respectively [15]. In their meta-analysis of the impact of elevated [CO_2_] on wheat grains, Broberg et al. [12] found a significant reduction in the concentration of the majority of minerals (Ca, Cd, Cu, Fe, Mg, Mn, P, S, and Zn), while B and Na were not significantly affected, and K was significantly increased (<2%). These meta-analytic results are in line with those from individual wheat FACE experiments [17,18,19,20,21]. Two minerals, Fe and Zn, are already deficient in the diets of hundreds of millions of people, and CO_2_-induced reductions in Fe and Zn have been reported in the edible parts of major crops [9,22,23] and are projected to have negative effects on human nutrition [24,25]. Furthermore, emerging evidence points to elevated [CO_2_] affecting nutrients beyond protein and minerals that are essential to humans, such as vitamins and carotenoids [26,27]. The decrease in mineral concentrations is notable in C_3_ plants but less so in C_4_ plants [9,23] and is consistent with differences in physiology; the simulation of carbohydrate production by elevated [CO_2_] is stronger in C_3_ plants, while reduced transpiration is present in both C_3_ and C_4_ plants.

During the last two decades, the air temperature has increased by 0.85 °C [28]. In fact, annual average minimum temperatures in Spain have increased over the last century by 1.5 °C, and by 0.6 °C during the last 25 years [29]. The most probable outcome of climate ensemble model projections foresees increasesof1.8 to 4.0 °C by the end of the 21st century (2090–2099) relative to the period 1980–1999. These numbers originate from the best estimate of greenhouse gas time series deduced from the six marker scenarios alone [30]. Heat stress is a major constraint to sustainable cereal production, with reductions in grain yield being associated with high temperatures during the reproductive or grain-filling stages in wheat [31,32] and rice [33,34,35]. High-temperature impacts on grain filling can vary enormously, depending on timing (days after anthesis) and duration. Both chronic moderately high temperatures (25–35 °C) and heat shocks (>35 °C) during the grain-filling phase are frequently associated with an increase in grain protein concentration in wheat [31,36,37] and rice [35,38]. Indeed, high temperature primarily impacts the accumulation of starch in wheat grain, with accumulation beginning earlier than under cooler temperatures, the duration of its accumulation also being reduced, and the result is a greater concentration of protein in the grain. Further, the duration of protein accumulation is reduced, while the rate of protein accumulation is substantially increased. In addition, leaves senesce before the heads mature, suggesting that high temperatures might enhance N remobilization from leaves and stems [39,40]. Moreover, the timing and duration of heat stress during grain filling have been shown to be important sources of variation in dough properties in wheat [41]. Grain protein and mineral composition are quality characteristics that can change due to high temperature, and they respond to changes in enzymes involved in starch and protein synthesis. Yang et al. [42] observed that the activity of glutamate synthase was enhanced by heat stress, while sucrose phosphate synthase, sucrose synthase, and soluble starch synthase were significantly decreased during grain filling. However, Monjardino et al. [43] found that protein concentration was negatively affected by heat stress during the early stage of endosperm development. They found that among the protein fractions, zeins are the most affected by heat stress. In fact, zein accumulation was repressed under high temperature rather than being degradedin the early developmental stages. In rice, elevated temperature also alters grain protein and mineral nutrient composition [35,44]. Ferreira et al. [36] showed that the total quantity of N per grain in wheat is generally little affected by the growing temperature but, due to the above-mentioned lower grain yield, the percentage of N on a dry weight basis rises under higher temperatures. Similar increases in the percentage of dry weight have been reported for wheat [45,46].

Increasing greenhouse gas emissions may also lead to rainfall reductions in the coming decades, which will increase the frequency and intensity of drought in the Mediterranean basin [47,48,49]. Climate change projections for the Mediterranean region indicate a precipitation decrease of 25–30% for the last decades of the 21st century [50]. Adding to that, the seasonality of rainfall is much more important. In fact, the expected shortage in Mediterranean rainfall should impact summer precipitation much more than winter precipitation. Mediterranean crop growth, however, is mainly driven by winter rain. Moreover, drought is considered one of the most important factors limiting crop yields around the world. Wheat crop responses to water scarcity depend on several factors, including plant development status, duration, and intensity of the stress and genetic variables [51]. Although rainfall during winter has been traditionally abundant and coincides with the lowest evapotranspiration rates, the occurrence of drought in winter during the early stages of the crop cycle has been recently reported [52].

This can further constrain wheat growth and thus final grain yield, mostly through a decrease in ear density and the number of kernels per unit crop area [53,54]. Grain yield reductions mediated by drought have been widely reported in wheat [55,56], and depending on the genotype, the reductions may reach up to 50%. The TGW is also reduced significantly, above 30% in droughted wheat [51,56,57]. Drought stress leads to reduced photosynthetic area and acceleration of leaf senescence during late grain filling in cereals, resulting in a shorter grain-filling period. In wheat, this smaller photosynthetic area and accelerated leaf senescence limit the amount of assimilates translocated to the grain, which implies reductions in grain yield [51]. Grain composition is also affected. Drought stress affects starch accumulation [51,58] more severely than N accumulation during grain filling, putatively influencing the conversion of sucrose into starch [59]. This tends to increase the grain protein concentration (expressed as % protein) in wheat [35,55,57] and rice [60]. In some cases, the opposite effect has been observed in wheat [61,62], possibly related to differences in stress levels and plant development status [63]. Knowledge of the effects of drought stress on grain mineral composition is scarce [7,64]. Crusciol et al. [60] explained the increase in rice grain N, Ca, Mg, Fe, and Zn concentration under rainfed conditions being due to a dilution effect because productivity was higher in irrigated than rainfed systems.

All the above-mentioned changes in grain composition linked to the changing environmental conditions are expected to have important implications for the nutritional quality of foods. During the last decade, different meta-analyses have characterized elevated [CO_2_] effects on crop yield and quality traits. However, comparatively little attention has been given to how other target environmental parameters such as temperature and drought will affect crop yield, and especially grain nutritional characteristics. Considering the economic and social importance of cereal crops and the impact of climate change not only on grain production but also on the nutritional value, this meta-analysis aims to provide an overview of the effects and interactions of multiple climate stressors, specifically high [CO_2_], drought and elevated temperatures, on the productivity and grain quality of C_3_ cereals.

## 2. Results

### 2.1. [CO_2_], Temperature and Drought Stress Effects on Grain Yield Components

**Figure 1:**
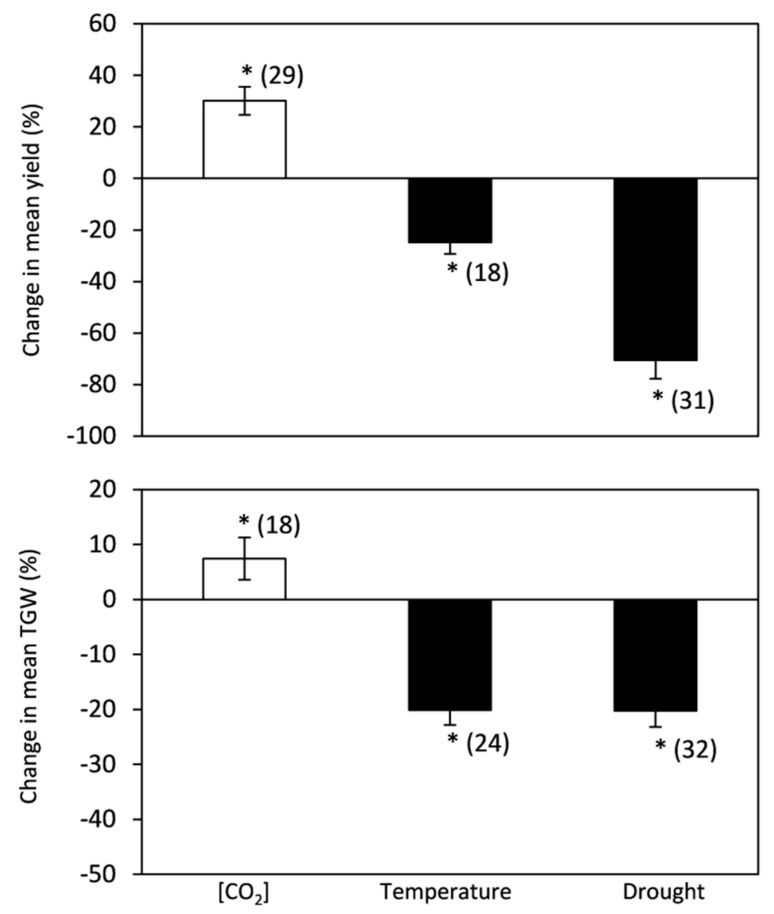
Change (%) in the mean of yield and TGW of plants grown under elevated [CO_2_], high temperature, and drought stress relative to the control. Data within parentheses indicate the number of observations. Error bars indicate the standard error of the mean. * indicates a statistically significant difference at *p* < 0.05.

The overall effect of elevated [CO_2_] on C_3_ crops resulted insignificant increases in grain yield and thousand grain weight (TGW) of30.10% and 7.41%, respectively (Figure 1). Nevertheless, a contrasting drastic loss in grain yield and TGW was observed under high temperatures and drought stress. Results presented in Figure 1 indicate that the heat and drought stress effects were similarfor TGW and recorded −20.17% and −20.29% reductions, respectively, but the negative effect of drought on cereal grain yield was larger than the effect of elevated temperatures (−70.53% vs. −24.85%).

### 2.2. [CO_2_], Temperature and Drought Stress Effects on Grain Quality

#### 2.2.1. Starch

**Figure 2:**
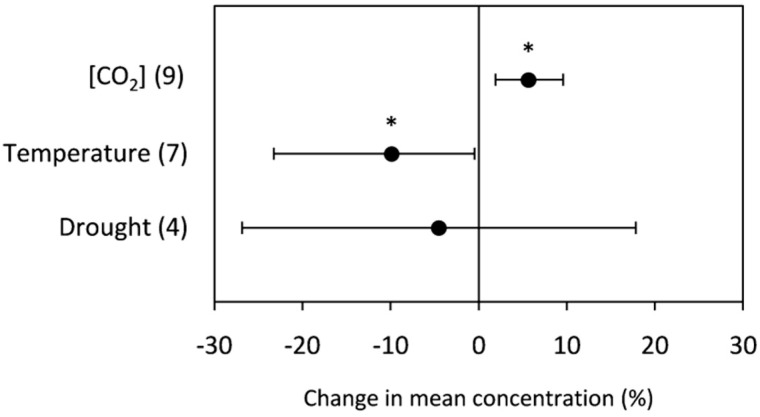
Change (%) in the mean concentration of grain starch of plants grown under elevated [CO_2_], high temperature, and drought stress relative to the control. Data within parentheses indicate the number of observations. Error bars indicate 95% CI. * indicates a statistically significant difference at *p* < 0.05.

In cereals grown under high [CO_2_], there was a significant increase in grain starch concentration (5.65%), whereas there was a significant decrease (−9.91%) under elevated temperature (Figure 2). Regarding water availability, there was no significant change as responses were sprayed in a broad range of both positive and negative changes (Figure 2).

#### 2.2.2. Total Protein

**Figure 3:**
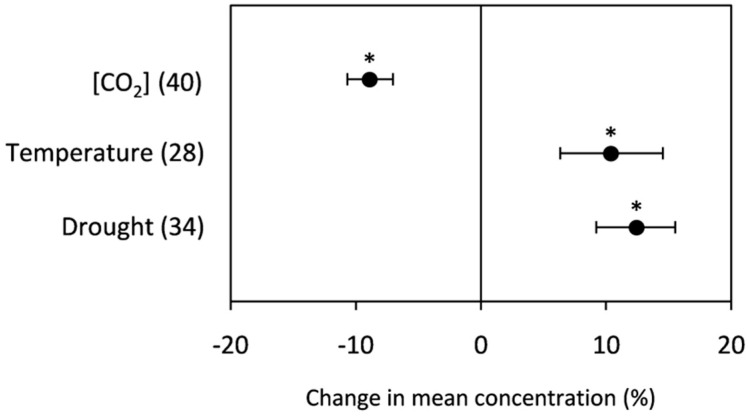
Change (%) in the mean concentration of grain total protein of plants grown under elevated [CO_2_], high temperature, and drought stress relative to the control. Data within parentheses indicate the number of observations. Error bars indicate 95% CI. * indicates a statistically significant difference at *p* < 0.05.

**Figure 4:**
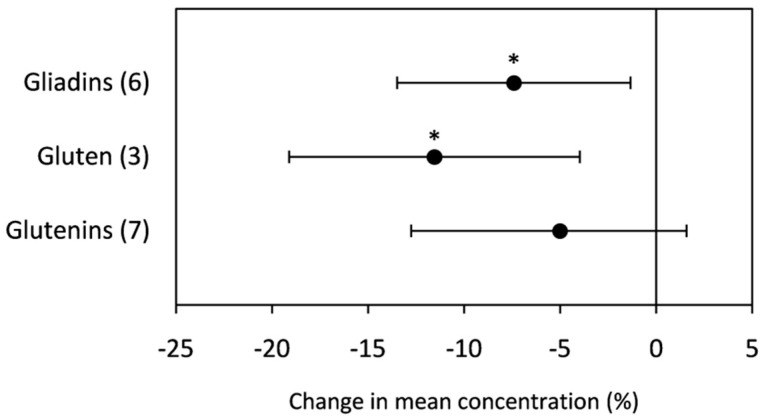
Change (%) in the mean concentration of grain gliadins, gluten, and glutenins of plants grown under elevated [CO_2_] relative to ambient level. Data within parentheses indicate the number of observations. Error bars indicate 95% CI. * indicates a statistically significant difference at *p* < 0.05.

Grain total protein concentration was negatively affected (−8.90%) by high [CO_2_]. However, it was significantly increased by temperature and drought (10.40% and 12.44%, respectively, as shown in Figure 3. Among the proteins that were studied, the gluten, gliadin, and glutenin concentrations were analyzed under elevated [CO_2_]. The grain gluten, gliadin, and glutenin concentrations presented in Figure 4 reveal a significant decrease in the gluten and gliadin concentrations (−11.54% and −7.41%, respectively). In contrast, rising [CO_2_] decreased the glutenin concentration, but it was not significant. Regarding the effects of drought and heat stress on these proteins, it was not possible to generate statistically powerful results due to the low amount of data (less than three repetitions).

#### 2.2.3. Mineral Composition

**Figure 5:**
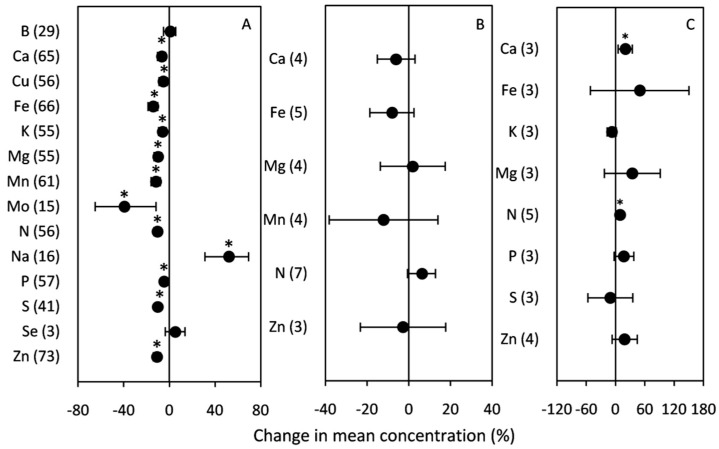
Change (%) in the mean concentration of grain minerals of plants grown under (**A**) elevated [CO_2_]; (**B**) high temperature; (**C**) drought stress relative to the control. Data within parenthesis indicate the number of observations. Error bars indicate 95% CI. * indicates statistically significant difference at *p* < 0.05.

The results presented in Figure 5 show an overall decrease in micro-macronutrients in C_3_ grains under elevated [CO_2_]. Across all the data, the mean change ranged between −4.70% (recorded for P) and −39.41% (recorded for Mo). The changes in B and Se were not significant. Among all the measured elements, only Na concentration increased significantly (52.05%) under high [CO_2_]. Heat stress had no significant effect on any of the grain mineral concentrations, and this could be due to data scarcity and small sample sizes leading to high data variability. Slight increases in Mg and N of1.91% and 6.31%, respectively, were recorded, whereas the Ca, Fe, Mn, and Zn concentrations were reduced. Regarding water scarcity, the data analysis showed distinct effects between minerals (Figure 5). In fact, drought stress induced an accumulation of Ca and N in grains and recorded a significant increase by 19.92% and 9.56%, respectively. However, no significant increase was obtained regarding Fe, Mg, P, and Zn concentrations. Under low water availability, S and K concentrations declined, but not significantly, by −10.43% and −7.59%, respectively.

## 3. Discussion

### 3.1. [CO_2_], Temperature and Drought Stress Effects on Grain Yield Components

Current scientific knowledge indicates that grain yield and quality will face serious challenges under the projected future climate. In line with previous papers [65,66,67], our meta-analysis shows that the predicted elevated [CO_2_] will increase crop grain production [8,17,68]. However, as noted by studies conducted over recent decades, it is essential to consider that the [CO_2_]-derived “fertilization” effect might decline or be eliminated when combined with stressful growth conditions, such as drought and temperature stress [69,70,71]. Moreover, in cereals such as wheat, increased grain yields have been associated with increases in the numbers of tillers and grains per spike rather than spike number or grain size [72,73]. The results of the current study have also revealed an association with an increase in the number of grains rather than their weight (larger increase in grain yield than TGW).

Both high temperature and drought negatively affected crop yield. Data analysis showed that yield was more markedly affected under drought than under heat stress conditions. Lower yields in stressed plants can be associated with (i) a shortened duration of the grain-filling period and/or (ii) a lowered photosynthetic rate during grain-filling. Dixit et al. [67] applied a crop simulation model to assess the impact of climate change on wheat production and found a loss of 15% in wheat grain yield in stressed plants, which was associated with a reduction in the number of days to reach grain maturity. Indeed, Mitchell et al. [74] attributed the direct negative effect of rising temperature on wheat yield to the temperature-dependent shortening of the phenological stages. Such decreases in the duration of grain filling would imply a shorter time available for accumulating resources for grain formation [31,46]. The time and duration of heat stress could cause different physiological responses in the plant, therefore, affect crop production. Many studies have reported that heat stress applied prior to anthesis negatively affects the grain yield of wheat due to many reasons [31,75]. High temperature accelerates leaf senescence and reduces post heading duration [75]. Adding to that, heat stress significantly reduces seed germination and negatively affects microspores and pollen cells, leading to non-functional florets or abortion of fertile florets and resulting in male sterility [76]. In fact, the decline in grain yields under high day temperatures was primarily caused by a reduction in the seed set percentage. Meanwhile, under high night temperature, the combination of decreased spikelet number per panicle, grain weight, and biomass production in addition to decreased seed set percentage contributed to the grain yield loss [77]. Altenbach et al. [51] reported that high temperature during anthesis promoted both grain shrinkage and a decrease in weight. Additionally, under heat stress conditions, plants tend to have a shorter grain-filling period, which reduces grain size and thousand kernel weight, while under drought conditions, plants tend to produce fewer grains per spikelet (and/or fewer tillers) [78]. This finding matches the TGW analyses stated above. In fact, heat stress and water scarcity showed similar effects on TGW in the current data analysis, suggesting that under drought conditions, the drastic decline inC_3_cereal yields is instead linked to a decrease in grain number produced per plant.

### 3.2. Effects of [CO_2_], Temperature and Drought Stress on Grain Quality

Another major consideration is the effect of climate change on grain quality. While crop breeding is already much more focused on yield traits, comparatively little attention has been given to grain quality traits. This is a matter of great concern because, as described in more detail below, environmental stress will affect the relative abundance of starch, protein, and minerals [9,79,80].

#### 3.2.1. Starch

Starch is the most abundant end-product of cereal growth and development, representing around 70% of the dry weight (*w*/*w*) of grains [81]. Rising [CO_2_] increases photosynthetic rates in C_3_ plants; increased carbohydrate translocation from the source (leaves and stems) to the sink (grains) is expected to increase the starch content in grains [82]. Indeed, the current data analysis has shown that growth under elevated [CO_2_] has a significant positive effect on the grain starch concentration, which contrasts with the non-significant results reported by Högy and Fangmeier [83] and Broberg et al. [12]. Fangmeier et al. [84] reported that elevated [CO_2_] significantly increased starch only for plants under high levels of N fertilizer.

Despite no significant effect due to drought, we revealed an overall decrease in grain starch concentration under drought stress. Worch et al. [85] observed that changes in endosperm starch content positively correlated with grain yield and concluded that grain starch content is one of the leading causes of reduced yield in crops subjected to drought conditions. This can be due to water deficit compromising both production of photoassimilates (source of carbon skeletons for the synthesis of starch) and the activity of enzymes involved in starch biosynthesis in the endosperm. Thus, the lower starch content observed in grains of genotypes subjected to water deficit could be correlated with the availability of reducing sugars [86].

Elevated temperature also negatively affected the starch concentration in grains. It has been reported that the reduction in starch concentration under high-temperature conditions is due to two factors; (i) shortening of the grain-filling period, which may reduce the duration of starch accumulation [51], and (ii) impairment of starch metabolism. While data for grains of plants exposed to high temperatures are scarce, Hawker and Jenner [87] and Keeling et al. [88] reported the inhibition of starch metabolism by high temperature (generally around 30 °C), possibly due to thermal denaturation negatively affecting the activity of starch synthase.

#### 3.2.2. Total Protein

Elevated [CO_2_] has been documented to reduce grain protein (or N) content in edible parts of crops [14,15,16]. In line with these earlier studies, the current meta-analysis showed that elevated [CO_2_] significantly decreased grain protein concentrations. This reduction has been associated with increased photosynthesis and accumulation of grain carbohydrates, leading to reductions in the amount of grain protein (due to a dilution effect) [17,35]. However, Goufo et al. [89] reported decreases in protein without associated increases in starch in grains of rice exposed to elevated [CO_2_]. Decreased protein concentrations in cereal grains under elevated [CO_2_] might be a consequence of reduced leaf protein concentrations in photosynthetic tissues, leading to decreased seed protein [84,90]. The suppression of nitrate assimilation by elevated [CO_2_] could be another contributor [91]. Our study also showed that there was a change in protein composition in grains of plants grown at elevated [CO_2_]. In line with the results of Wieser et al. [92] and Högy et al. [17], gluten, gliadins, and glutenin concentrations decreased under increasing [CO_2_]. Differences in the amounts and proportions of gluten protein fractions and types have significant effects on dough mixing and rheological characteristics. One of the most important characteristics for baking quality is bread volume, which has been strongly correlated with crude protein, total gluten proteins, and glutenin macropolymers [4,93]. Consequently, a reduction in bread quality can be expected due to the higher sensitivity of gluten fractions to elevated [CO_2_].

Grain protein content is sensitive to environmental conditions and controlled by a number of factors, particularly the duration and rate of grain filling and the availability of assimilates, which are negatively affected in crops subjected to stressful growth conditions [94,95]. In contrast to elevated [CO_2_], we found that high temperatures increased the grain protein concentration by 10.4%, which could be attributed to greater remobilization of shoot-derived protein. The grain protein concentration is expressed as a percentage of grain dry mass, which alongside the lower size and weight of the affected grains (also detected in our meta-analysis), would contribute to them having lower carbohydrate levels and consequently higher grain protein [96]. We note that the increase in grain protein concentration (10.4%) is almost the same as the decrease in grain starch concentration (−9.9%), suggesting that starch depletion increases the relative content of total protein.

Drought affects plant phenology and physiology. Water scarcity has been previously described as reducing photosynthetic rates, shortening the grain-filling period [11,97], and accelerating leaf senescence after anthesis. We detected significant increases in grain total protein associated with low water availability. Bhullar and Jenner [98] reported that during the grain-filling period, drought stress hinders the conversion of sucrose into starch but has a milder effect on protein biosynthesis. Our findings did not corroborate Bhullar and co-workers’ conclusions. As mentioned before, the fact that the grain starch concentration was not significantly affected by drought would discard the lower carbohydrate level as a factor that induces increased grain protein content. Singh et al. [7] observed that together with lower rates of carbohydrate accumulation in the grain of plants subjected to drought, the increase in flour protein was mainly due to higher rates of grain N accumulation. The present meta-analysis supports this assertion because grain N concentration was affected by drought. Adding to that, the increased grain protein concentration under drought could be explained by the shortened maturation time common to stress conditions, which tends to favor protein over starch accumulation in cereal grains [99]. Drought, among other stresses, accelerates the translocation of senescence-inducing resources (including amino acids) from leaves to seeds during grain filling. Several studies have demonstrated that the contribution of reserve mobilization to the final grain yield is higher under stressful conditions than relatively well-irrigated conditions [100,101,102].

#### 3.2.3. Mineral Composition

The present study showed that elevated [CO_2_] leads to an impoverishment of macro/microelements in grains. Moreover, there is a variation among minerals in the magnitude of the reductions, and this supports previous results [17,18,20,26,103]. In fact, only the Na concentration was significantly increased, with surprisingly few studies having investigated this element in relation to the effect of [CO_2_], and so there is little background information to explain this trend. Basically, most studies have focused on the main minerals that affect human health, such as Fe, Zn, P, K, and Ca, and have underlined a common decline in these minerals under rising [CO_2_]. With respect to our results, the concentrations of Zn, Fe, S, Ca, Mg, P, Mn, K, and Mo were significantly decreased. Such reductions have been associated with increased production of spikes and grains that translates into a grain nutrient-dilution effect, diminishing the nutritional value. Furthermore, by reducing transpiration (linked to stomatal closure due to long-term exposure to elevated [CO_2_]), high [CO_2_] can reduce the mass flow in the soil toward roots, which diminishes the availability of mobile minerals in the rhizosphere [14]. While carbohydrate dilution should lower all other nutrients in plant tissues evenly [104], other effects of elevated [CO_2_] on plant physiology are not evenly distributed among the minerals. For example, reduction in transpiration and elevated biosynthesis affect some minerals more than others. This means that the stoichiometry of plants exposed to elevated [CO_2_] should “differ not only in C:(other elements) ratios but also in the ratios among other elements (e.g., C:N, N:P, and P:Zn should be different)” [14]. Indeed, Loladze’s meta-analysis of over 7500 pairs of observations from studies of elevated [CO_2_] published over 30 years (1984–2014) showed a significant reduction in foliar Mg (and N, P, K, Ca, S, Fe, Zn, and Cu) but not the Mn content in C_3_ plants, and underlying biochemical mechanisms responsible for the increased Mn:Mg ratio have been proposed [105].

Changes in the elemental composition in grains are also detected under heat and drought stress. Previous studies suggested that both stress factors tend to increase mineral concentrations (including Fe, N, S, Zn, K, and P). However, the low number of reports means that there is relatively large uncertainty about the magnitude of the increase. The observed increase in grain protein and N concentrations (and the concomitant decrease in starch) under elevated temperature means that there is more N per unit of starch [106]. In addition, Fe and Zn tend to increase under drought. Although water plays a significant role in mineral uptake and later mobilization within the plant, with these processes decreasing during water stress, our meta-analysis agrees with Ge et al. [107] reporting that soil drought stress improved transport mechanisms and/or routes for some minerals, such as Fe and Zn, leading to increased grain concentrations of these elements. Moreover, according to other studies [107,108], the increase in the levels of Fe and Zn may be related to the more efficient remobilization of these nutrients from leaves to grains. However, according to other authors [109], the increase in Fe and Zn concentrations is linked to sink strength at the single grain level. More specifically, Miller et al. [109] observed in maize how the mineral content in drought-sensitive genotypes (which produced lower numbers of grains than the tolerant ones) was higher than in fully watered plants. According to this explanation, the increase in nutrients in the grains may be related to the number of grains formed, with each grain being a specific sink [86]. Furthermore, as we mentioned above, heat and drought cause a decrease in the number and size of cereal grains, which suggests that there might be a concentration effect due to the smaller grains [110].

## 4. Materials and Methods

### 4.1. Data Search and Selection Criteria

To find relevant studies related to the issue of the current meta-analysis, literature searches of primary research in published peer-reviewed journal sources were conducted from Google, Web of Science, and Scopus in June 2017. To search the literature, the following keywords were used: grain yield, cereal, high [CO_2_], elevated temperature, drought stress, climate change, and C_3_ grain quality. More than 150 papers were found, but 78 articles were selected according to the following criteria: (i) the article studies the effect of at least one climate parameter, including [CO_2_], temperature, and drought, (ii) the article contains at least one response variable from the following list: grain yield, thousand grain weight (TGW), starch, total protein, gluten, glutenins, gliadins, and a set of minerals (Al, N, B, Ca, Cd, Co, Cr, Cu, Fe, K, Mo, Mg, Mn, Na, Ni, P, Pb, S, Se, Si, and Zn). The most abundant C_3_ species that are reported in the literature are wheat, rice, and barley. All papers included in this meta-analysis were published between 1990 and 2019 (Table A1). The study is based on comparing plants grown at elevated [CO_2_] (550–900 ppm) using Open Top Chamber (OTC) facilities or in the field using Free-Air-CO_2_-Enrichment (FACE) systems with those grown at ambient [CO_2_] (currently at ca. 400 ppm). Studies comparing different ranges of temperature, from ambient (10–25 °C) to elevated temperature (28–37 °C), and two levels of irrigation (limited irrigation or well-watered) are also included in the current report. Response means of plants grown under the different environmental conditions stated previously were taken from tables. The time of occurrence of stress during the crop cycle and the duration of stress applied differ among the studies, as indicated in Table S1. Most studies reported that treatments were maintained until the end of the experiments, when the plants reached maturity.

### 4.2. Data Analysis

All the data described above were organized in an Excel datasheet pairwise (control and experimental value) for each experimental factor ([CO_2_], temperature, water) (Table S1). The datasheet was loaded into and analyzed in RStudio v1.1.456 [111]. For the effect size metric, we used the natural log of the response ratio, lnR = ln(HF/LF), where LF and HF are reported mean nutrient concentrations at low and high treatment, respectively, with the treatment being any of the three climate factors considered in this study (CO_2_, temperature, or water). The log response ratio eliminates asymmetry between percentage decreases limited to 100% and unlimited percentage increases; it is a standard approach for analyzing elevated [CO_2_] and other ecological studies [112]. After performing statistical analyses, all the results were back-transformed to regular percentage changes using the formula: (exp(lnR) − 1)*100%.For estimating the 95% confidence intervals for the mean effect size, a non-parametric test, namely bootstrapping with 999 replacements, was used for sample sizes of seven or more (i.e., when seven or more independent studies reported any given nutrient concentration at low and high treatments) [27]. The advantage of this approach is that it does not require the distribution of effect sizes to be normal. However, for the confidence intervals to be accurate, they can be applied only for sufficiently large sample sizes (>7). For sample sizes <7, we had a choice of discarding the data completely, which would result in the loss of potentially valuable information, or making a normality assumption and applying a parametric method. We chose the latter for sample sizes of 3 to 7. No confidence intervals were derived for sample sizes of two or less. In all cases, unweighted methods were used, with each study having equal weight.

## 5. Conclusions and Perspectives

This study highlights that while current and near-future environmental conditions will severely affect cereal yield, the nutritional value of cereal grain will also be affected.

It seems that within the three factors related to climate change investigated, the rise in atmospheric [CO_2_] is possibly the one more detrimental and difficult to face because elevated [CO_2_] will impact grain quality traits all over the world while the impacts of the increase in temperature and the decrease in water availability will be localized or easy to counterbalance. In fact, although the increase in [CO_2_] might promote yield enhancement and starch accumulation through higher rates of photosynthesis, the grains of these plants will have lower concentrations of total proteins and minerals, leading to reduced baking quality and deficient nutritional value. On the other hand, even if both high temperature and drought severely decrease crop yields, the available data shows that grain quality will be differentially affected. Heat stress will negatively affect grain starch concentration due to depleted starch biosynthesis metabolism and shortening of the grain-filling period, but it might increase total proteins and N concentration. Regarding water availability effects, grain yield could be conditioned by the final starch concentration of affected plants. Adding to the increase in the Fe and Zn concentrations, we found that total protein concentrations are significantly increased, which is probably due to a dilution effect on starch and the accelerated reserve remobilization from source to sink to compensate for the nutrient uptake deficit that results from low soil water content. According to numerous climatic models, precipitation patterns are expected to change in the future with more frequent drought events in semiarid and arid regions but, it is also predicted that in other regions, precipitation will likely increase. Therefore, while drought and elevated temperature can be potentially mitigated (by increasing irrigation, planting crops at higher altitudes within a given latitude, or displaced to cooler and wet latitudes within a country), the effect of rising [CO_2_] is present at all latitudes and will act independently of where crops will be established. Hence, [CO_2_]-induced reductions in grain quality would be much more challenging to mitigate.

Our study highlights the fact that within the context of the present and near-future environments, it is crucial to increase crop yield through the development of stress-adapted cultivars. While the current breeding programs and agricultural incentives are almost exclusively yield-based, breeding for improved cereal quality can meaningfully improve the nutritional status of humanity. For this purpose, a better understanding of how environmental growth conditions (such as elevated temperature, drought, etc.) affect grain yield and nutritional parameters of cereals will help developing more nutrient-dense crops. Adding to that, exploring genetic diversity and variability of major crops is needed to discover genotypes more resilient to ongoing climate change.

## Data Availability

Data is contained within the article or supplementary material.

## Supplementary Materials

The following is available online at https://www.mdpi.com/article/10.3390/plants10061052/s1, Table S1: The collected data and information used in the meta-analysis.

## Appendix A

**Table A1:** List of papers and species used for the data analysis of each environmental factor.

Papers	Species
**High [CO_2_]**	
[92]	*Triticum durum* L.
[113]	*Triticum aestivum* L.
[17]	*Triticum aestivum* L.
[114]	*Triticum aestivum* L.
[115]	*Triticumaestivum* L./*Hordeumvulgare*
[26]	*Oryza sativa* L.
[116]	*Oryza sativa* L.
[73]	*Triticum aestivum* L.
[22]	*Triticumdurum* L.*/Oryza sativa* L.
[117]	*Triticum aestivum* L.
[20]	*Triticum aestivum* L.
[118]	*Triticum aestivum* L.
[119]	*Triticum aestivum* L.
[120]	*Triticum aestivum* L.
[121]	*Triticum aestivum* L.
[122]	*Triticum aestivum* L.
[84]	*Triticum aestivum* L.
[123]	*Triticum aestivum* L.
[124]	*Triticum aestivum* L.
[125]	*Triticum aestivum* L.
[126]	*Triticum aestivum* L.
[127]	*Triticum aestivum* L.
[74]	*Triticum aestivum* L.
[128]	*Triticum aestivum* L.
[129]	*Triticum durum* L.
[38]	*Oryza sativa* L.
[80]	*Triticum durum* L.
[18]	*Triticum durum* L.
[19]	*Triticum aestivum* L.
[130]	*Triticum aestivum* L.
[131]	*Oryza sativa* L.
[132]	*Triticum aestivum* L.
[133]	*Triticum aestivum* L.
[134]	*Triticum durum* L.
[35]	*Oryza sativa* L.
[135]	*Oryza sativa* L.
**Drought stress**	
[136]	*Triticum aestivum* L.
[57]	*Triticum durum* L.
[137]	*Triticum aestivum* L.
[138]	*Triticum aestivum* L.
[139]	*Triticum durum* L.
[140]	*Triticum durum* L.
[141]	*Triticum aestivum* L.
[55]	*Triticum durum* L.
[62]	*Triticum aestivum* L.
[80]	*Triticum durum* L.
[60]	*Oryza sativa* L.
[61]	*Triticum aestivum* L.
[129]	*Triticum durum* L.
[126]	*Triticum aestivum* L.
[56]	*Triticum aestivum* L.
[142]	*Triticum aestivum* L.
[37]	*Triticumaestivum* L., *Triticumdurum* L.
[143]	*Triticum aestivum* L.
[144]	*Triticum aestivum* L.
[145]	*Triticum aestivum* L.
[51]	*Triticum aestivum* L.
**Heat stress**	
[36]	*Triticum durum* L.
[51]	*Triticum aestivum* L.
[32]	*Triticum aestivum* L.
[146]	*Triticumaestivum* L., *Triticumdurum* L.
[59,147]	*Triticum aestivum* L.
[45]	*Triticum aestivum* L.
[148]	*Triticumaestivum* L., *Triticumdurum* L.
[31]	*Triticum durum* L.
[145]	*Triticum durum* L.
[39]	*Triticum aestivum* L.
[74]	*Triticum aestivum* L.
[149]	*Triticum aestivum* L.
[46]	*Triticum aestivum* L.
[150]	*Triticumaestivum* L., *Triticumdurum* L.
[151]	*Triticum aestivum* L.
[37]	*Triticumaestivum* L., *Triticumdurum* L.
[152]	*Triticum aestivum* L.
[153]	*Triticum aestivum* L.
[41]	*Triticum aestivum* L.
[154]	*Triticum durum* L.
